# Modeling Monthly Variation of *Culex tarsalis* (Diptera: Culicidae) Abundance and West Nile Virus Infection Rate in the Canadian Prairies

**DOI:** 10.3390/ijerph10073033

**Published:** 2013-07-22

**Authors:** Chen-Chih Chen, Tasha Epp, Emily Jenkins, Cheryl Waldner, Philip S. Curry, Catherine Soos

**Affiliations:** 1 Large Animal Clinical Sciences, Western College of Veterinary Medicine, University of Saskatchewan, 52 Campus Drive, Saskatoon, SK S7N 5B4, Canada; E-Mails: tasha.epp@usask.ca (T.E.); cheryl.waldner@usask.ca (C.W.); 2 Department of Veterinary Microbiology, Western College of Veterinary Medicine, University of Saskatchewan, 52 Campus Drive, Saskatoon, SK S7N 5B4, Canada; E-Mail: emily.jenkins@usask.ca; 3 Saskatchewan Ministry of Health, 3475 Albert Street, Regina, SK S4S 6X6, Canada; E-Mail: pcurry@health.gov.sk.ca; 4 Environment Canada, Science & Technology Branch, 115 Perimeter Road, Saskatoon, SK S7N 0X4, Canada; E-Mail: catherine.soos@ec.gc.ca

**Keywords:** West Nile virus, *Culex tarsalis*, geographic information system, generalized linear mixed model, environmental variables, Canadian prairie

## Abstract

The Canadian prairie provinces of Alberta, Saskatchewan, and Manitoba have generally reported the highest human incidence of West Nile virus (WNV) in Canada. In this study, environmental and biotic factors were used to predict numbers of *Culex tarsalis* Coquillett, which is the primary mosquito vector of WNV in this region, and prevalence of WNV infection in *Cx. tarsalis* in the Canadian prairies. The results showed that higher mean temperature and elevated time lagged mean temperature were associated with increased numbers of *Cx. tarsalis* and higher WNV infection rates. However, increasing precipitation was associated with higher abundance of *Cx. tarsalis* and lower WNV infection rate. In addition, this study found that increased temperature fluctuation and wetland land cover were associated with decreased infection rate in the *Cx. tarsalis* population. The resulting monthly models can be used to inform public health interventions by improving the predictions of population abundance of *Cx. tarsalis* and the transmission intensity of WNV in the Canadian prairies. Furthermore, these models can also be used to examine the potential effects of climate change on the vector population abundance and the distribution of WNV.

## 1. Introduction

Since the introduction of West Nile virus (WNV) into eastern North America in 1999 [1], WNV has become an endemic disease in the most of southern Canada, especially in the Canadian prairie provinces of Alberta, Saskatchewan, and Manitoba, which have had the highest human infection rate in Canada. Of the 2,315 cases in Canada in 2007, more than 98% occurred in these three prairie provinces [2]. Saskatchewan alone accounted for over half the human cases reported in Canada. Research focusing on the drivers of WNV occurrence in the prairie ecosystem is needed to inform WNV control and public health interventions.

West Nile Virus is primarily transmitted and amplified among local avian fauna and ornithophilic mosquito vectors, with occasional spillover into mammalian populations through mosquito blood feeding [3,4,5,6]. Therefore, the WNV infection rate in mosquito vectors is commonly utilized as an indicator of pathogen transmission intensity [7,8] and has been demonstrated to be a better indicator for WNV activity than surveillance of dead or infected birds [9]. Surveillance of mosquito infection rate also provides sufficient lead time for intervention and management of mosquito borne diseases [7,9].

Although WNV has been isolated from at least 59 mosquito species in North America, only a small portion including mosquitoes belonging to the genera *Culex* (Diptera: Culicidae) have been shown to be competent vectors [5]. *Culex tarsalis* Coquillett is considered to be the main vector of WNV in the Canadian prairies [3,10]. It is one of the most efficient WNV vectors evaluated in the laboratory studies [4] and the predominant species in the Canadian prairies during the summer WNV season [3]. Several biological features of *Cx. tarsalis* also facilitate the transmission of WNV in the enzootic cycles. *Culex tarsalis* can vertically transmit WNV to its offspring [11], it takes several blood meals, and it produces multiple generations per season [3]. Furthermore, it is known to feed on both avian and mammalian hosts and plays the role of the “bridge vector” which transmits WNV out of its enzootic cycle to humans and other mammalian species [12].

Environmental variables such as temperature, precipitation and habitat type influence both *Cx. tarsalis* population abundance and its WNV infection rate [13,14,15,16]. In addition, environmental factors may influence seasonal and spatial overlap among key hosts in the sylvatic amplification cycles. For instance, drought-induced congregation of mosquitoes and birds on shrinking wetland habitats may enhance the transmission of WNV [17]. Therefore, understanding how environmental and biotic factors influence abundance of mosquito population and WNV infection rate in mosquitoes is important in predicting the risks of WNV.

The Canadian prairies are at the northern limit of WNV distribution in the Western hemisphere. Climate and habitat suitability for *Cx. tarsalis* determine the distribution of this mosquito and WNV in the prairie provinces [18]. Future alterations in climate or habitat might expand the current spatial and temporal distribution of *Cx. tarsalis* and WNV [19,20]. A predictive model using environmental factors as the primary explanatory variables could be applied to evaluate the potential effects of climate change. 

Many studies have been conducted to clarify the effects of environmental and biotic factors on the risk of WNV and predict the distribution of WNV in North America since its incursion. However these models usually cannot be applied to predict risks in other regions, particularly when those regions have different ecological dynamics or primary vector species [21]. Previous work had evaluated the effects of climate factors on the distribution of WNV by constructing a weekly model to predict the weekly variation of WNV infection rate in the Canadian prairies [22]. The objectives of this present study were to clarify how environmental and biotic factors affect the abundance and WNV infection rate of *Cx. tarsalis* on a monthly scale and compare this to the weekly model. In addition, due to the limitation of time scale of the climate change dataset, it was necessary to construct a monthly model fitted with the monthly dataset, the finest time scale for evaluating the effects of climate change [23].

## 2. Materials and Methods

### 2.1. Mosquito Data

WNV infection in pooled female *Cx. tarsalis* was determined using reverse transcription polymerase chain reaction (RT-PCR) [24,25]. Data on mosquito trap locations, abundance as the number of *Cx. tarsalis* per trap night, and WNV infection from across the prairie provinces were obtained from May to September for 2005 to 2008 from the Public Health Agency of Canada (PHAC). The original data were supplied to PHAC from Alberta Environment, Manitoba Public Health and Healthy Living, and Saskatchewan Ministry of Health.

**Figure 1:**
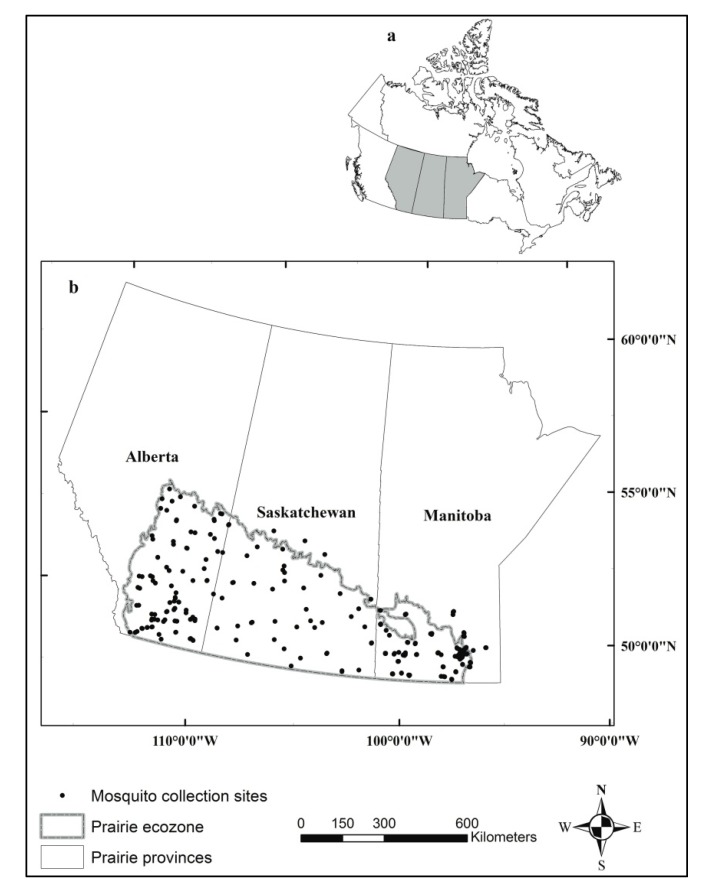
Distribution of mosquito sampling sites in the Canadian prairies provinces of Alberta, Saskatchewan, and Manitoba for the period from 2005 to 2008. (**a**) Location of prairie provinces (grey spot) in Canada. (**b**) The distribution of sampling sites across the Canadian prairies ecozone.

Mosquito sampling sites were distributed within the different health regions across the southern half of the prairie provinces (Figure 1). The new standard miniature light traps with photocell controlled CO_2_ release (Model 1012-CO_2_; John W. Hock Company, Gainesville, FL, USA) were used for mosquito sampling. The mosquito collection period generally began in late May and lasted until the end of August (in Manitoba, the collection period ended the first week of September). During each week, the trap was operated for one night at each of the mosquito collection sites in Alberta and Manitoba, but for one to four nights per week at Saskatchewan collection sites. Monthly mean of *Cx. tarsalis* abundance, individuals per sampling night, was estimated for each collection site.

WNV infection rate (per 1,000 *Cx. tarsalis*) was computed using PooledInfRate (version 3.0), A Microsoft^®^ Excel plug-in [26] by Maximum Likelihood (ML-IR) and minimum infection rate (MIR) methods [26,27]. WNV infection rates in the mosquito population were usually low, especially in the early transmission season. In this period, estimations of arbovirus infection rate in mosquitoes with values of zero were commonly recorded. Therefore, to achieve reasonable detection probability, large mosquito sample size for screening was required [28,29,30]. Gu and Novak [30] suggested that for a medium detection probability of 0.5, 693 mosquitoes are required. 

In addition, we also found extremely high infection rates in some records where a positive result was obtained from pooled test which was comprised of only a few mosquitoes. Excluding observations from our dataset with low *Cx. tarsalis* sample sizes in the late season of WNV (usually in the early September) might also remove the records with high infection rate; however, female *Cx. tarsalis* are usually inseminated and preparing for hibernation in this period in which they do not take a blood meal [3] and the risk of WNV transmission is considered to be low. Based on these findings, we excluded observations with samples less than 100 female *Cx. tarsalis* per site per week from the analysis to prevent potential outliers and incorrect estimation of WNV infection rate resulting from small sample size [30]. The monthly mean WNV infection rate in *Cx. tarsalis* was calculated for each collection site.

### 2.2. Land Cover

The land cover dataset was derived from the Advanced Very High Resolution Radiometer (AVHRR) sensor operating on board the United States National Oceanic and Atmospheric Administration satellites. AVHRR Land Cover Digital Data was downloaded from Natural Resources Canada. Although the satellite image was taken in 1995, the land use in this area was considered to have remained relatively stable until the start of the study period [31]. 

Data for the prairie provinces were extracted and converted to a single 1 km^2^ GIS raster layer. Eleven different types of land cover categories were included in the original dataset. According to habitat utilization by *Cx. tarsalis* [18], land cover was simplified into forest including deciduous, transitional coniferous and mixed forests, water, barren land, agricultural land including cropland and rangeland, and urbanized area. To analyze the association between land cover, *Cx. tarsalis* population, and infection rate, we used 20 km radius buffer zones around collection sites, defined according to the flight distance of *Cx. tarsalis* [10,32] to determine the percentage of primary composition of land cover type within the buffer zones.

### 2.3. Weather Data

Daily mean temperature, daily maximum temperature, daily minimum temperature, and daily total precipitation were downloaded from the National Climate Data and Information Archive, Environment Canada. The daily weather datasets were used to create various predicting variables (Table 1). Daily maximum and minimum temperature were used to calculate the accumulative degree days [33] by the single sine method [34] for current month, and two and three months accumulative degree days (Table 1). We also created the monthly mean degree days predictors by using monthly mean maximum and minimum temperature [35]. For estimating the monthly mean degree days, the low temperature threshold for WNV amplification in *Cx. tarsalis* was set as 14.3 °C [15]. Finally, to determinate possible nonlinear effects of weather variables on *Cx. tarsalis* abundance and WNV infection rate, this study also created second order polynomial variables using the centered value of temperature and precipitation. Weather predictors of each climate station were interpolated by the *inverse distance weighted* method to create prairie-wide climate raster layers in ArcGIS. There was a total of 473 climate stations located in the Canadian prairies which were evenly distributed across the study area.

**Table 1:** Descriptions of variables used in both *Cx. tarsalis* abundance and infection rate models and relationships between explanatory variables based on the Pearson correlation and principal component analysis. Variables with the same arabic number indicated that the Pearson correlations are larger than 0.8 or have factor loading larger than 0.6 in each component of principal component analysis.

Variables	Correlation (>0.8)			PCA component (Factor loading >0.6)		Variables description
	LMM	GLMM		LMM	GLMM	
Monthly mean temperature	1	1		1	1	Monthly mean temperature of the month of mosquito data collection
1 month lagged mean temperature	2	2		2	2	1 month lagged mean monthly temperature
2 month lagged mean temperature	2	2		2	2	2 months lagged mean monthly temperature
3 months mean temperature	2	2		2	2	Including mosquito collection month, and previous 1 and 2 months
Winter mean temperature		4		3	3	From December to February
Monthly mean degree days	3	3		3	3	Monthly mean degree day of the mosquito data collection month
2 months total of monthly mean degree days	3	3		3	3	Created by summing the monthly mean degree days of the month of mosquito data collection and previous month
3 months total of monthly mean degree days	-	3		-	3	Created by summing the monthly mean degree days of the month of mosquito data collection, previous one and two months. Not applied in the LMM
Mean temperature fluctuations	3	3, 4		3	3	Monthly mean maximum temperature minus monthly mean minimum temperature
1 months accumulative degree days	1	1		1	1	The accumulative degree days of data collection month
2 months accumulative degree days	2	2		2	2	The accumulative degree days of data collection month and previous months
3 months accumulative degree days	-	2		-	2	The accumulative degree days of data collection month and previous one and two months. Not used in the LMM
1 month lagged mean precipitation	-	4		-	4	1 month lagged monthly mean daily total precipitation.
Monthly total precipitation						Monthly total precipitation
1 month lagged total precipitation		4		4	4	1 month lagged monthly total precipitation
2 month lagged total precipitation				2	2	2 month lagged monthly total precipitation
Total precipitation of previous year				4	4	Annual total precipitation of previous year
3 months total precipitation				4	4	The total precipitation of mosquito collection month, and previous one and two months

LMM: Linear mixed model for predicting *Cx. tarsalis* abundance; GLMM: Generalized linear mixed model for predicting WNV infection rate in *Cx. tarsalis*; “-”: variable is not used in the model construction.

### 2.4. Data Analysis

Counts of *Cx. tarsalis* per trap site per night were transformed by ln(y + 1) to normalize the data distribution prior to analysis. Models to predict *Cx. tarsalis* abundance using environmental factors were constructed using linear mixed model (PROC MIXED, SAS ver. 9.2, SAS Institute, Cary, NC, USA).

A generalized linear mixed model with a log link function (PROC GLIMMIX, SAS ver. 9.2, SAS Institute, Cary, NC, USA) was then used to develop a model for *Cx. tarsalis* infection rate prediction. A negative binomial distribution was chosen in the *Cx. tarsalis* infection rate model according to a preliminary analysis where the formula “Pearson Chi-Square divided by degrees of freedom (DF)” value was close to one. This formula was used to evaluate overdispersion.

The Pearson correlation test was used to test for multi-collinearity between explanatory variables. If the correlation between any pair of variables was larger than 0.8, the more significant variable (lower −2 log likelihood) was chosen for further model construction [36]. We also conducted principal component analysis for determining the relationship between explanatory variables and compared the results with those of the Pearson correlation. Components selected were based on eigenvalues (>than 1) and the cumulative percentage of variance accounted for by the components (>than 80%). Factor loading larger than 0.6 of each variables was considered to be highly correlated with the component [37]. Health regions where mosquito collection traps were located were used as a random effect for both *Cx. tarsalis* abundance and infection rate models. The variable parameters were estimated by the restricted maximum likelihood and Gauss-Hermite quadrature method for *Cx. tarsalis* abundance and infection rate models, respectively [38].

### 2.5. Model Selection and Validation

Explanatory variables selected in the models of *Cx. tarsalis* abundance and WNV infection rate were based on the Wald test with p threshold value set at 0.05 [38,39] The backward stepwise method was adopted for variable selection. The AICc (AIC value corrected for finite sample sizes) values and AICc weights were used to assess the models fit and select the best fitted model [38,39,40]. Four-fifths of the records were randomly selected from the dataset for model construction and the remaining one-fifth of the records were used for model validation. Standardized residuals and standardized Pearson residuals were estimated for the validation dataset of *Cx. tarsalis* abundance and infection rate models, respectively. Values of Root Mean Square Error (RMSE) were calculated for training and validation datasets to validate and compare the predictability of the model on each dataset. Moran’s *I* test was used to test the spatial autocorrelation of residuals for the final models. 

The final model of WNV infection rate in *Cx. tarsalis* was applied to create maps of the predicted WNV infection rate in the Canadian prairies using ArcGIS 10 (Environmental System Research Institute, Redlands, CA, USA). To compare the predicted *Cx. tarsalis* infection rate and actual human WNV incidence in the prairie provinces, maps of human WNV incidence (cases per 100,000 individuals) were created for the entire transmission season (May-September) for each health region for 2005 to 2008. Human WNV case data was retrieved from prairie provincial governmental websites [41,42,43]. 

## 3. Results

### 3.1. Descriptive Statistics

**Figure 2:**
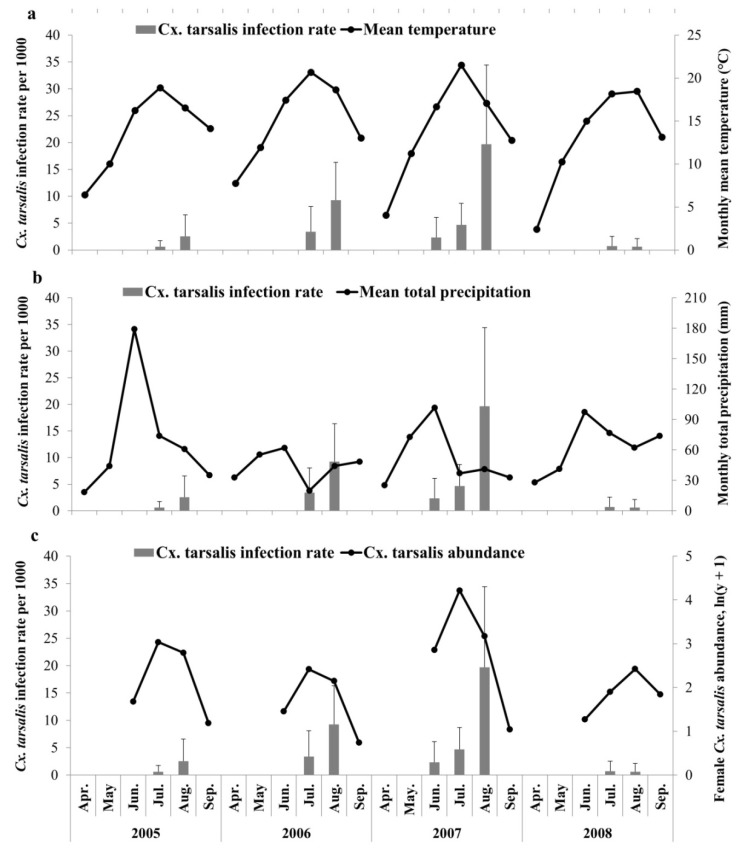
Temporal trends of (**a**) Monthly mean temperature (unit 1°C). (**b**) Monthly total precipitation (unit 1 mm). (**c**) Monthly mean abundance of *Cx. tarsalis*, ln(y+1) transformed, compared to monthly mean WNV infection rate in female *Cx. tarsalis* in the Canadian prairies. Error bar indicates the standard deviation of mean *Cx. tarsalis* infection rate.

Out of 309 mosquito collection sites, 96 were in Alberta, 38 in Saskatchewan and 175 in Manitoba (Figure 1). Mosquito collections were conducted from late May to early September and the highest observed mean abundance of *Cx. tarsalis* was in July (for 2005 to 2007) or August (for 2008) (Figure 2). The highest observed mean *Cx. tarsalis* infection rates were in August for 2005 to 2007; there was no obvious trend observed in 2008 (Figure 2).

**Figure 3:**
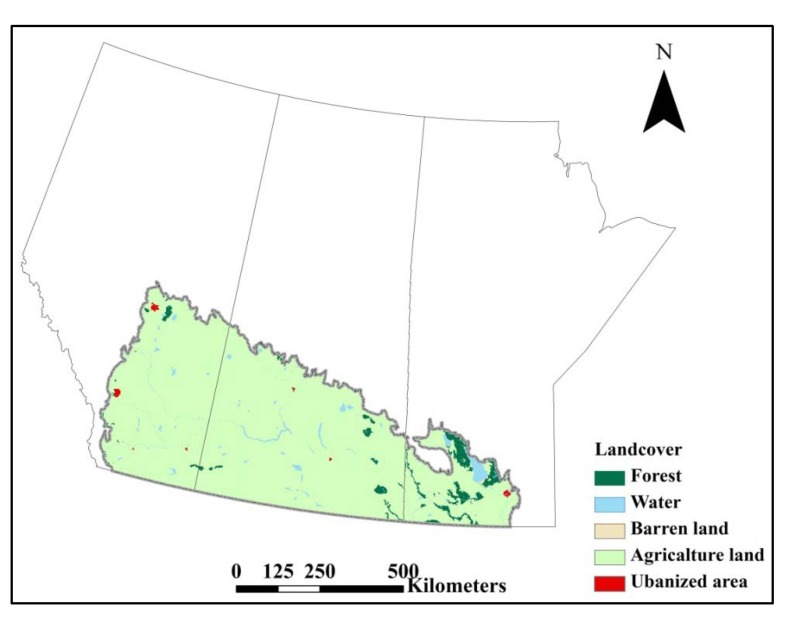
Distribution of land cover types in the Canadian prairies.

July was the warmest month with temperature around 20 °C. The mean temperature in July 2007 was higher than other years, ranging from one to three Celsius degree, and 8.6% higher than mean temperature in July of study period. The range of monthly temperature fluctuation was from 6.3 to 20 °C in the Canadian prairie ecozone. In 2007, total precipitation in May was highest compared to other years with a mean temperature closer to 2006 but higher than 2005 and 2008 in the Canadian prairies (Figure 2). Agriculture land cover predominated the buffered zones of mosquito collection sites; 94.3% of sampling sites had agriculture land as the primary land cover type. Mean percentage of agriculture land was 86.1% in each buffer zone. The other two primary land cover types identified were forest (2.21%) and water (3.52%) (Figure 3). 

### 3.2. Constructed Models

Based on the results of Pearson correlation and principal component analysis, explanatory variables could be grouped into four highly correlated components (Table 1). The cumulative variances explained by these four components of principal component analysis were 84.6% and 86.1% for *Cx. tarsalis* abundance and infection rate models, respectively.

In the final *Cx. tarsalis* abundance model, increased mean monthly temperature, 1 month lagged mean temperature, total precipitation, and temporal lags of precipitation from 1 to 2 months were significantly associated with increased *Cx. tarsalis* abundance, while an inverse association between forest land cover and *Cx. tarsalis* abundance was observed (Table 2).

In the final WNV infection rate model, we found that increasing *Cx. tarsalis* abundance, and 1 month lagged temperature were associated with increased WNV infection rate, while one month lagged mean precipitation, 3 months total precipitation, and water land cover were inverse associated with infection rate (Table 3).

**Table 2:** Estimated coefficients of explanatory variables in the constructed models of *Cx. tarsalis* abundance. Single variable indicates the explanatory variables which are assessed individually. Final model represents the final fitted model with the lowest AICc value. Full model is model fitted with all created explanatory variables.

Variables	Single variable			Final model			Full model	
	Coef.	95% CI		Coef.	95% CI		Coef.	95% CI
*Intercept*				−3.48 *	−4.05 to −2.91		−3.93 *	−4.6 to −3.25
*Weather*								
Monthly mean temperature	0.25 *	0.22 to 0.26		0.22 *	0.2 to 0.25		0.22 *	0.19 to 0.25
1 month lagged temperature	0.08 *	0.07 to 0.1		0.07 *	0.05 to 0.09		0.06 *	0.04 to 0.09
Winter mean temperature	−0.04 *	−0.06 to −0.01					−0.03 *	−0.06 to −0.01
Monthly mean degree days	0.28 *	0.23 to 0.33					0.032	−0.04 to 0.1
Monthly total precipitation	−0.003 *	−0.004 to −0.001		0.0033 *	0.002 to 0.005		0.0032 *	0.002 to 0.005
1 month lagged precipitation	0.006 *	0.005 to 0.007		0.0042 *	0.003 to 0.005		0.0037 *	0.002 to 0.004
2 month lagged precipitation	0.005 *	0.004 to 0.006		0.0033 *	0.002 to 0.004		0.003 *	0.002 to 0.005
*Land cover* *^1^*								
Forest	−0.48 *	−0.91 to −0.04		−0.54 *	−0.9 to −0.17		−0.59 *	−0.95 to −0.22
Water	−0.11 *	−0.47 to −0.26					0.03	−0.28 to 0.34

Coef.: estimated variable coefficient; * P < 0.05; ^1^ agriculture land was used as a reference group.

**Table 3:** Estimated coefficients of explanatory variables in the constructed models of WNV infection rate. Single variable indicates the explanatory variables which are assessed individually. Final model represents the final fitted model with the lowest AICc value. Full model is model fitted with all created explanatory variables.

Variables	Single variable			Final model			Full model	
	Coef.	95% CI		Coef.	95% CI		Coef.	95% CI
*Intercept*				−2.26 *	−4.47 to −0.05		−1.64	−5.64 to 2.37
*Cx. tarsalis* abundance	0.16 *	0.03 to 0.30		0.55 *	0.31 to 0.79		0.58 *	0.28 to 0.87
*Weather*								
Monthly mean temperature	−0.14 *	−0.2 to −0.08					−0.04	−0.18 to 0.10
1 month lagged temperature	0.25 *	0.21 to 0.29		0.32 *	0.22 to 0.41		0.32 *	0.21 to 0.42
Winter mean temperature	0.23 *	0.06 to 0.40					0.01 *	−0.13 to 0.15
3 months total of monthly mean degree days	0.20 *	0.16 to 0.24		−0.10 *	−0.2 to −0.01		−1.10	−0.21 to 0.002
Monthly total precipitation	−0.015 *	−0.02 to −0.01					−0.01	−0.02 to 0.003
1 month lagged mean precipitation	−0.48 *	−0.56 to −0.39		−0.27 *	−0.36 to −0.18		−0.43 *	−0.62 to −0.24
2 month lagged total precipitation	0.003	−0.001 to 0.01					−0.01	−0.02 to 0.003
3 months total precipitation	−0.085	−0.11 to 0.06		−0.05 *	−0.08 to −0.02		0.013	−0.06 to 0.08
*Land cover* *^1^*								
Forest	−1.3 *	−1.84 to −0.76					−0.43	−1.27 to 0.41
Water	−1.31 *	−2.8 to −0.182		−1.52 *	−2.56 to −0.47		−1.61 *	−2.85 to −0.38

Coef.: estimated variable coefficient; *: P < 0.05; ^1^: agriculture land was used as a reference group.

In addition, we found the inverse association between the 3-months total of monthly mean degree days and WNV infection rate when the lagged mean temperature was controlled. Time lagged mean temperature was the distorter variable which distorted the coefficient of the 3-months total of monthly mean degree days from a positive association when this variable was tested alone to negative association.

Second-order polynomial variables of temperature and precipitation were not significantly associated with *Cx. tarsalis* abundance and infection rate. The RMSE of training and validation dataset was 0.97 and 1.0, respectively. Both RMSE values were close to one and close to each other which indicated the accuracy and precision of model predictability. There was no significant spatial autocorrelation detected for residuals of *Cx. tarsalis* infection rate by Moran’s *I* test (p = 0.65). 

**Figure 4:**
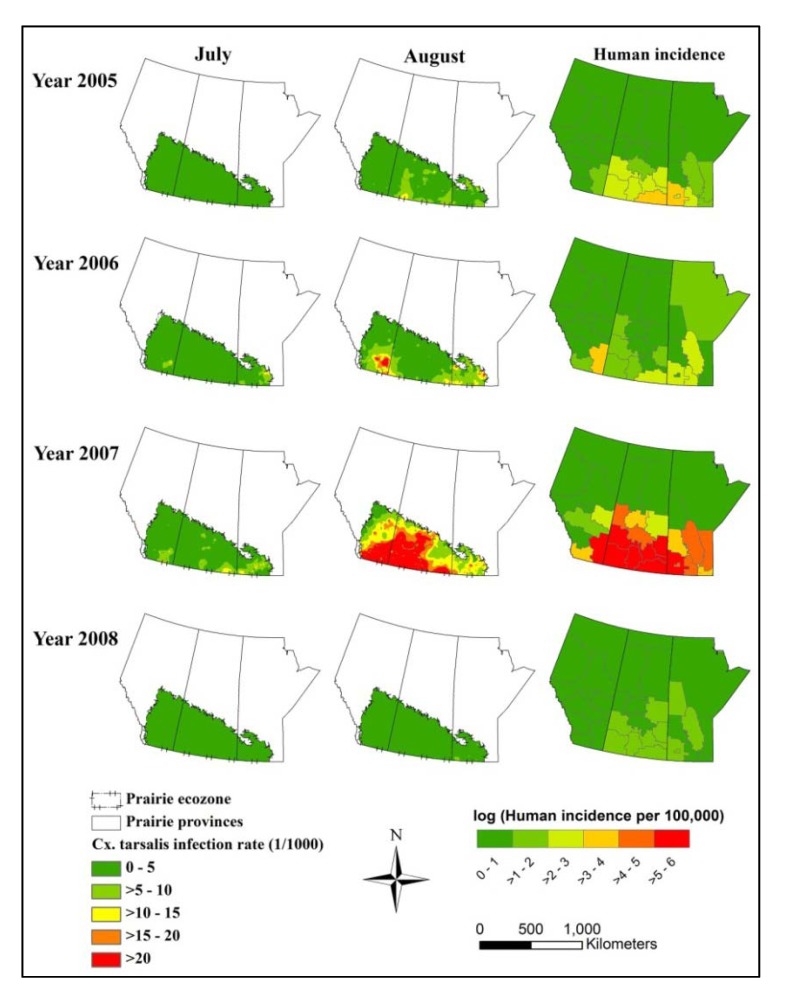
Maps of predicted WNV infection rate in female *Cx. tarsalis* per 1,000 in July and August 2005–2008 in the Canadian prairies and log transformed human incidence (cases per 100,000 individuals) for each health region in the entire WNV transmission season.

We used the *Cx. tarsalis* infection rate of 20 per 1,000 as a criterion to represent the high risk area in our predictive maps based on the mean mosquito infection rate in August 2007, a major epidemic period of WNV in the Canadian prairies. In our predictive maps, the southern part of the Canadian prairies was generally at higher risk, especially in southeast Alberta, southwest to southeast Saskatchewan and southwest Manitoba (Figure 4).

## 4. Discussion

This study analyzed the effects of environmental drivers on *Cx. tarsalis* abundance and infection rate of WNV from 2005 to 2008. Most of the geographic regions at highest risk based on predicted WNV infection rate in *Cx. tarsalis* were consistent with the distribution of human WNV cases between 2005 and 2008. The slight differences between the distribution of mosquito infection rate and human incidence were expected, and can potentially be explained by social and economic factors, population density, risk perception, mosquito control programs, and the intensity of human case detection in different health regions. However, mosquito infection rate as an indicator for early season forecasting or predicting of WNV human incidence has been previously demonstrated [9]. Mosquito infection rate was also influenced by variations in climate factors. Of particular interest here, understanding the relationship between climate factors and mosquito infection on monthly scale could be adopted to predict WNV activity under different climate change scenarios [23]. 

In this study, more explanatory variables were evaluated and compared with our previously published weekly model [22]. The variables associated significantly with WNV infection rate in this study were similar to the weekly model; however, on the monthly time scale, we found an inverse relationship between mean degree days and WNV infection rate when time lagged temperature was controlled. In addition, we demonstrated the effects of land cover composition in a 20 km radius buffer zone on the abundance of *Cx. tarsalis* and WNV infection rate. We also found a better predictability of monthly model than weekly model based on the values of Root Mean Square Error (data not shown).

In both *Cx. tarsalis* abundance and WNV infection rate models on the monthly time scale, increasing temperature and lagged temperature significantly increased *Cx. tarsalis* abundance and infection rate. The mean temperature in May and June, 2007 was higher than 2005 and 2008, with the highest mean temperature in July during study period. High environmental temperature with the antecedent highest precipitation in May and *Cx. tarsalis* abundance in June might have contributed to the outbreak of WNV in 2007. Increasing environmental temperature shortens the maturation time required for *Cx. tarsalis* and the extrinsic incubation period of virus. Furthermore, it also accelerates the gonotrophic cycle and affects mosquito survival. In combination, these relationships influence virus transmission by increasing the contact rate between mosquito and host [15,33].

This study found the distorted effect of time lagged mean temperature on the 3 months total of monthly mean degree days for the model of WNV infection rate. Increasing 3 months total of monthly mean degree days can decrease the WNV infection rate in *Cx. tarsalis* when the variable of time lagged mean temperature was controlled. In addition, the Pearson correlation and principal component analysis revealed that mean temperature fluctuations, and 3 months total of monthly mean degree days were highly correlated. These variables were all estimated based on the maximum and minimum temperature and indicated the degree of temperature fluctuation. These findings indicated that increasing temperature fluctuation could decrease the *Cx. tarsalis* infection rate in the environment with similar time lagged mean temperature. The effect of high temperature fluctuation has been proposed to limit the midgut infection of flaviviruses in mosquitoes by preventing the virus from entering the midgut epithelial cells or limiting initial replication of virus in these midgut cells [44]. Adverse effect of high temperature fluctuations have also been observed in the transmission of dengue virus by *Aedes aegypti* [45] and western equine encephalomyelitis by *Cx. tarsalis* [46,47].

The probable contact rate between an infected competent vector and a susceptible host is essential for maintaining the enzootic cycle of an arbovirus in a geographic area, although herd immunity of host population might dampen the transmission of WNV [48]. A minimum threshold of vector and susceptible host interaction is needed to allow for virus transmission [49]. Therefore, population abundance of suitable competent vectors is usually an indicator of the prevalence of pathogen occurrence [49,50,51]. A positive association has been demonstrated for other arboviruses such as Japanese encephalitis, western equine encephalitis and St. Louis encephalitis [49,52,53]. Furthermore, abundance of *Cx. tarsalis*, *Cx. p. quinquefasciatus*, *Cx. pipiens* and *Cx. restuans* has also been used as indicators to predict human WNV risk in different regions [54,55,56].

In contrast to temperature, the influence of precipitation on the enzootic cycle of WNV is paradoxical. Increased precipitation is generally believed to create standing water suitable for mosquito breeding and increase mosquito population and the risk of vector borne disease. This reason likely accounts for why precipitation and time lagged precipitation were positively associated with mosquito abundance in our study. In 2007, total precipitation in May was highest compared to other years with the mean temperature closer to 2006 but higher than 2005 and 2008 in the Canadian prairies. The high precipitation might have contributed to the explosive occurrence of *Cx. tarsalis* in June. Other researchers have demonstrated that preceding droughts can increase the incidence of WNV in human population in the western United States [57] and the Canadian prairies [58]. Potential explanations for the effects of precipitation on WNV incidence include drought induced decreases in mosquito competitors or predators and increase the abundance of *Cx. tarsalis*, increased congregation of susceptible avian hosts and mosquito vector on dwindling wetland habitat, and changes in the composition of the avian host community [17,59,60]. In our study, precipitation was positively associated with *Cx. tarsalis* abundance, similar to studies in California and the northern Great Plain habitat in South Dakota [14,16] but an inverse association between precipitation and WNV infection rate in *Cx. tarsalis* was observed. These findings suggest that other factors, such as the aggregation of competent hosts and *Cx. tarsalis* [59], or alterations to composition of the avian community might better explain the effects of precipitation on WNV infection rate in *Cx. tarsalis* in the Canadian prairies [61].

Nonlinear relationships between climate factors and arthropod vectors are commonly observed. For instance, Reisen *et al.* [14] found that spring *Cx. tarsalis* population was positively correlated with temperature in winter and spring, whereas summer abundance was correlated negatively with spring temperature and not correlated with summer temperature in California. A unimodal relationship between precipitation and WNV incidence was demonstrated in the northern Great Plains and the optimal total precipitation from May to July was approximately 200 mm [62]. In our study, all the second-order polynomial variables were not associated with *Cx. tarsalis* abundance or infection rate. Total precipitation in May to July of 2005 to 2008 ranged from 79 to 332 mm and observed high risk areas had the lowest precipitation values. The warmest summer mean temperature was around 20 °C in July (Figure 2), which was lower than the mean temperature of 30 °C for the same month in California [56]. Low summer environmental temperatures across the Canadian prairies could be the main reason for our findings. In this temperature range, the development rate of *Cx. tarsalis* and the replication rate of WNV in the *Cx. tarsalis* are linearly related with environmental temperature [13,15].

Ezenwa *et al.* [61] found a negative association between WNV infection rate among *Culex* mosquitoes and wetland coverage. Wetland area has been positively associated with bird species diversity and thus it is possible that this represents an example of the dilution effect, in which increased bird diversity led to overall decreases in the mosquito infection rate [61,63]. In our models, *Cx. tarsalis* abundance was not significantly different between wetland and agriculture as the primary composition of land cover types; however, a significant negative association was found between wetland and *Cx. tarsalis* infection rate. This result reflects the effect of possible underlying factors, such as the differences of bird species composition between different land cover types and also indicates that vector abundance alone was not sufficient to predict the intensity of WNV occurrence.

## 5. Conclusions

Our study clarifies the relationship between environmental factors and the abundance of *Cx. tarsalis* and infection rate of WNV in the Canadian prairies. The observed association between environmental temperature and WNV infection rate could provide sufficient time to predict WNV occurrence and initiate disease control and public health interventions. In addition, warmer temperature and increased variability in precipitation are already being observed and are projected to accelerate in the Canadian prairies [64]. Predictive monthly models for vector-borne diseases are critical tools for public health and wildlife management in a future of rapid climate change. These models could be adopted to assess the effects of changing climate conditions on WNV in the Canadian prairies [23]. 
